# Ornithine decarboxylase antizyme inhibitor 2 (AZIN2) is a signature of secretory phenotype and independent predictor of adverse prognosis in colorectal cancer

**DOI:** 10.1371/journal.pone.0211564

**Published:** 2019-02-15

**Authors:** Tuomas Kaprio, Tiina Rasila, Jaana Hagström, Harri Mustonen, Petri Lankila, Caj Haglund, Leif C. Andersson

**Affiliations:** 1 Department of Surgery, Helsinki University Central Hospital, Helsinki, Finland; 2 Research Programs Unit, Translational Cancer Biology, University of Helsinki, Helsinki, Finland; 3 Department of Surgery, Lahti Central Hospital, Lahti, Finland; 4 Department of Pathology, Haartman Institute, University of Helsinki and HUSLAB, Helsinki, Finland; Columbia University, UNITED STATES

## Abstract

Ornithine decarboxylase (ODC) is the rate-limiting enzyme of polyamine synthesis. The two ODC antizyme inhibitors (AZIN1) and (AZIN2) are regulators of the catalytic activity of ODC. While AZIN1 is a regulator of cell proliferation, AZIN2 is involved in intracellular vesicle transport and secretion. There are no previous reports on the impact of AZIN2 expression in human cancer. We applied immunohistochemistry with antibodies to human AZIN2 on tissue micro- arrays of colorectal cancers (CRC) from 840 patients with a median follow-up of 5.1 years (range 0–25.8). The 5-year disease-specific survival rate was 58.9% (95% Cl 55.0–62.8%). High AZIN2 expression was associated with mucinous histology (p = 0.002) and location in the right hemicolon (p = 0.021). We found no association with age, gender, stage, or histological tumor grade. High tumor expression of AZIN2 predicted an unfavorable prognosis (p<0.0001, log-rank test), compared to low AZIN2 expression. Cox multivariable analysis identified AZIN2 as an independent factor of an unfavorable prognosis in CRC. The strongest AZIN2 expression was seen in invasive tumor cells having morphological features of epithelial-mesenchymal transition (EMT). Induction of EMT in HT-29 CRC cells lead to upregulated expression of endogenous AZIN2. Given that AZIN2 is a regulator of vesicle transport and secretion, we overexpressed human AZIN2 cDNA in T84 CRC cells, and found strongly enhanced accumulation of CD63-positive exosomes in the culture medium. These findings indicate that AZIN2 expression is a signature of EMT-associated secretory phenotype that is linked to an adverse prognosis in CRC.

## Introduction

Colorectal cancer (CRC), with over one million new cases every year, is one of the three most common cancers worldwide, and its incidence is rising. Early detection, radical surgery, and adjuvant chemotherapy are important for clinical outcome. Stage of disease at diagnosis is the most crucial factor for predicting patient outcome; 40% of the patients have localised disease and another 40% have regional disease. [1]

Today adjuvant therapy is the standard care for Stage III patients, giving an absolute 10% increase in 5-year overall survival. Of the patients with stage II disease, 80–85% are cured by surgery alone. T4-stage, high histological grade, vascular invasion, tumor obstruction, bowel perforation, and inadequate lymph node resection have been considered a reason for adjuvant therapy, even though the prospective data supporting this concept are limited. It would be important to identify those Stage II patients who benefit from postoperative treatment. [2]

Polyamines are organic polycations that are involved in the regulation of a variety of cellular functions, ranging from proliferation and malignant transformation to differentiation and apoptosis and their cellular levels are regulated by biosynthesis, up-take, catabolism and excretion [3]. Ornithine decarboxylase (ODC) is the rate-limiting enzyme of polyamine synthesis. High ODC activity is typically found in rapidly proliferating normal and malignant cells and in cancerous tissue. ODC is a transcriptional target of the c-myc oncogene [4], but ODC itself also displays oncogenic properties. Over-expression of human ODC cDNAin mouse NIH3T3 fibroblasts induced malignant transformation [5] with the ability for tumor growth in athymic mice [6]. Given the impact of ODC on cellular processes, its activity is stringently regulated both at transcriptional and post-translational levels [7]. A large proportion of ODC is bound as catalytically inactive monomers to proteins called antizymes (AZ) [8].

Antizyme inhibitors (AZIN) are proteins that are highly homologous to ODC but without catalytic activity. The AZINs are antagonists of AZs, which they bind with higher affinity than ODC, and thereby release sequestered ODC to form catalytically active homodimeric molecules. [8]

Two forms of mammalian AZINs have been described. The first one, now called AZIN1, was reported in 1982 by Fujita et al [9]. AZIN1 is involved in normal and malignant cell growth. Elevated expression of AZIN1 is typical to cancer cells. Amplification of the AZIN1 gene has also been reported in different human neoplasms, including cancer of the breast, prostate, lung and liver. [10]

The second form of human AZIN, originally named ODC-p and now called AZIN2, was originally identified and cloned in 2001 by Pitkänen et al [11]. While expression of AZIN1 is mainly seen in proliferating cells, the highest levels of AZIN2 are found in many terminally differentiated cells such as neurons and megakaryocytes [12]. AZIN2 has been found to be involved in the regulation of intra-cellular vesicle transport [13] and mast cell degranulation [14]. Immunohistochemistry revealed high physiological AZIN2 expression in tissues with secretory activity and in those with abundant vesicle traffic, such as brain neurons and exocrine glands [12].

Immunohistochemical staining of sections of colon cancers with antibodies to human AZIN2 revealed its elevated expression in the invasive cells of the tumor fronts. This observation prompted us to investigate a tissue micro-array material consisting of 840 colorectal cancers in order to evaluate the prognostic role of AZIN2 expression, and its association with clinicopathological parameters. Based on our finding that AZIN2 is a regulator of vesicle transport and mast cell degranulation, we used a human colon cancer cell line to study the impact of AZIN2 expression on *in vitro* release of exosome-like material.

Exosomes are small cell-derived vesicles that are released from normal and malignant cells, and they are found in virtually all body fluids. They carry a variety of bioactive molecules, including proteins, lipids, RNA and DNA, which may be taken up by acceptor cells, thereby comprising a system of intercellular communication. Exosomes derived from cancer cells have been shown to contribute to the formation of pre-metastatic niches, as well as to stimulate metastatic spread and tumor angiogenesis. Elevated plasma levels of circulating tumor-derived exosomes have been reported to correlate with poor prognosis and shorter survival time in CRC. [15]

## Materials and methods

### Patients

The study population comprised 840 consecutive CRC patients operated on in 1983–2001 at the Department of Surgery, Helsinki University Hospital (HUH). The Finnish Population Register Centre provided the follow-up vital-status data needed to compute survival statistics, and Statistics Finland provided the cause of death for all those deceased. The median age of the patients at diagnosis was 66 years, with a median follow-up of 5.1 years (range 0–25.8). The 5-year disease-specific survival (DSS) rate was 58.9% (95% Cl 55.0–62.8%).

This study complies with the Declaration of Helsinki and was approved by the Surgical Ethics Committee of Helsinki University Hospital (Dnro HUS 226/E6/06, extension TMK02 §66 17.4.2013). The National Supervisory Authority of Welfare and Health gave us permission to use tissue samples without the individual informed consent of the patients in this retrospective study (Valvira Dnro 10041/06.01.03.01/2012).

### Cell cultures and induction of EMT and stable transfections

The human colorectal carcinoma cell lines, HT-29, T84, LS174T, and the lung carcinoma cell line A549 (obtained from ATCC Manassas, VA, USA) were cultured in RPMI (Sigma-Aldrich, Saint Louis, MO, USA) supplemented with 10% (v/v) inactivated fetal bovine serum (Gibco, Thermo Fisher, Waltham, MA, USA), 1 mM _L-_glutamine (Honeywell Fluka, Thermo Fisher, Waltham MA,USA), 50 mg/ml penicillin, 50 mg/ml streptomycin and G418 (800 microg/ml) at 37°C in an atmosphere of 5% CO_2_ in air.

We induced EMT in HT-29 cells [16]. The cultures were serum-starved for 5 h before addition of recombinant human TGF-β1 (Sigma; 10 ng/ml) and human TNF-α (R&D Systems; 10 ng/ml) in medium supplemented with one percent FBS. After 48 h of cultivation, the cell morphology was monitored and the samples were harvested for qRT-PCR and western blotting.

The T84 cell line was stably transfected with pcDNA3 (Invitrogen, Carlsbad, CA, USA) encoding human AZIN2 cDNA [17]. Control cells were transfected with the empty vector.

### RT-PCR, qRT-PCR and primers

To examine transcription of the AZIN2 gene in HT-29 and T84 cells, total RNA was extracted using the TRI REAGENT -RNA/DNA/Protein isolation reagent (Molecular Research Centre, Inc., Cincinnati, OH, USA) according to the manufacturer’s instructions. Total RNA (1 ug) was reverse- transcribed with the High Capacity RNA-to cDNA Kit (Applied Biosystems, Foster City, CA, USA). cDNA was used as a template for quantitative real-time PCR analysis using a Maxima SYBR Green/ROX qPCR Master Mix (Thermo Fisher) and a LightCycler II instrument (Roche Diagnostics, Mannheim, Germany). The primers used for real-time analysis were: AZIN2 (forward) 5’-AGGGGCCAAAGTGAGATTTG-3’ and (reverse) 5’-CTTGGCAATGATGCTGACTG-3’; GAPDH (forward) 5’-GGTGAAGGTCGGAGTCAAC-3’ and (reverse) 5’-CAAATGAGCCCCAGCCTTC-3’.

#### Isolation of exosomes / exosome-like material

Exosome-like particles (here called exosomes) were purified from culture supernatants of T84 cells stably over-expressing AZIN2, and control cells by differential ultracentrifugation as described by Théry et al. [18]. Equal numbers of over-expressing and control cells were grown for 48 h in total of 200 ml exosome-production medium (complete medium depleted of FBS-derived exosomes by overnight ultracentrifugation and sterile filtration). Conditioned medium was purified from contaminating cell material by serial centrifugation (10 min at 300 g, 20 min at 2,000 g, and 30 min at 10,000 g). Exosomes were pelleted from the purified conditioned medium by ultracentrifugation at 100,000 g for 70 min at 4°C (SW32Ti, Beckman Coulter Inc., FL, USA). The pellets were resuspended in a large volumes of PBS, centrifuged at 100,000g for 70 minutes at 4°C, and resuspended in 400 ul PBS, centrifuged at 100,000 g for 60 min at 4°C (Beckman Airfuge). Finally exosome preparation was resuspended in 50 ul PBS with protease inhibitors.

Lysates of the cells that produced the conditioned medium were simultaneously prepared and analyzed in parallel with the exosomes. For immunoblot analysis, the cells were lysed in buffer (50 mM Hepes, pH 7.0, 150 mM NaCl, 10% glycerol, 1% Triton X-100, 1.5 mM MgCl_2_, 1mM EGTA, 100 mM NaF, 10 mM Na_4_P_2_O_7_ with protease inhibitors). The amount of proteins present in the exosome preparations and in the total cell lysates was quantified by the Bradford assay (Bio-Rad, Hercules, CA, USA). We analysed the isolated exosomes / exosome-like material by western blotting using antibodies to the tetraspanin proteins CD63 and CD9 that are commonly used exosome markers [19].

### Western blot analysis

The proteins were resolved by 12% SDS-PAGE and transferred to hydrophobic Immobilon PVDF membranes (Millipore, MA, USA). The membranes were blocked with Odyssey blocking buffer (LI-COR Biosciences), incubated with the primary antibodies, followed by incubation with goat anti-rabbit Alexa Fluor-680 (Invitrogen Molecular Probes) and donkey anti-mouse IRDye 800CW (LI-COR Biosciences, Lincoln, NE, USA) antibodies (both 1:10 000). An Odyssey Infrared Imager (LI-COR-Biosciences) was used for visualization.

### Antibodies

To detect AZIN2 expression we used rabbit antibody raised against a peptide (STRDLLKELTLGASQATT) representing the amino acids 18–35 of AZIN2. The production and validation of the specificity of the rabbit AZIN2 antibody called K3 has been described earlier [14,20]. Other primary antibodies used were mouse monoclonal anti-human GAPDH (G8795), mouse monoclonal anti-human CD63 (SAB4700215) and rabbit polyclonal anti-human CD9 (SAB4503606), all from Sigma-Aldrich; HSP90α/β (sc-13119) was from Santa Cruz Biotechnology.

### Tissue microarray

Formalin-fixed and paraffin-embedded tumor samples were obtained from the archives of the Department of Pathology, HUH. An experienced pathologist marked representative areas of the tumor samples on hematoxylin- and eosin-stained tumor slides. Three 1.0-mm-diameter punches from each sample were mounted in recipient paraffin blocks with a semiautomatic tissue microarray instrument (Beecher Instruments, Silver Spring, MD,USA) as described [21].

### Immunohistochemistry

Freshly cut 4-μm sections of tumor tissue microarray blocks were fixed on slides and dried at 37°C for 12–24 h. After deparaffinization in xylene and rehydration through a gradually decreasing concentration of ethanol to distilled water, the slides were treated in a PreTreatment module (Lab Vision Corp., Fremont, CA, USA) in antibody-specific buffer for 20 min at 98°C for antigen retrieval. The sections were stained in an Autostainer 480 (Lab Vision) by the Dako REAL EnVision Detection system, Peroxidase/DAB+, Rabbit/Mouse (Dako, Glostrup, Denmark). The tissues were incubated with the K3 primary antibody (dilution 1:700) for one hour at room temperature.

### Scoring of samples

**Fig 1:**
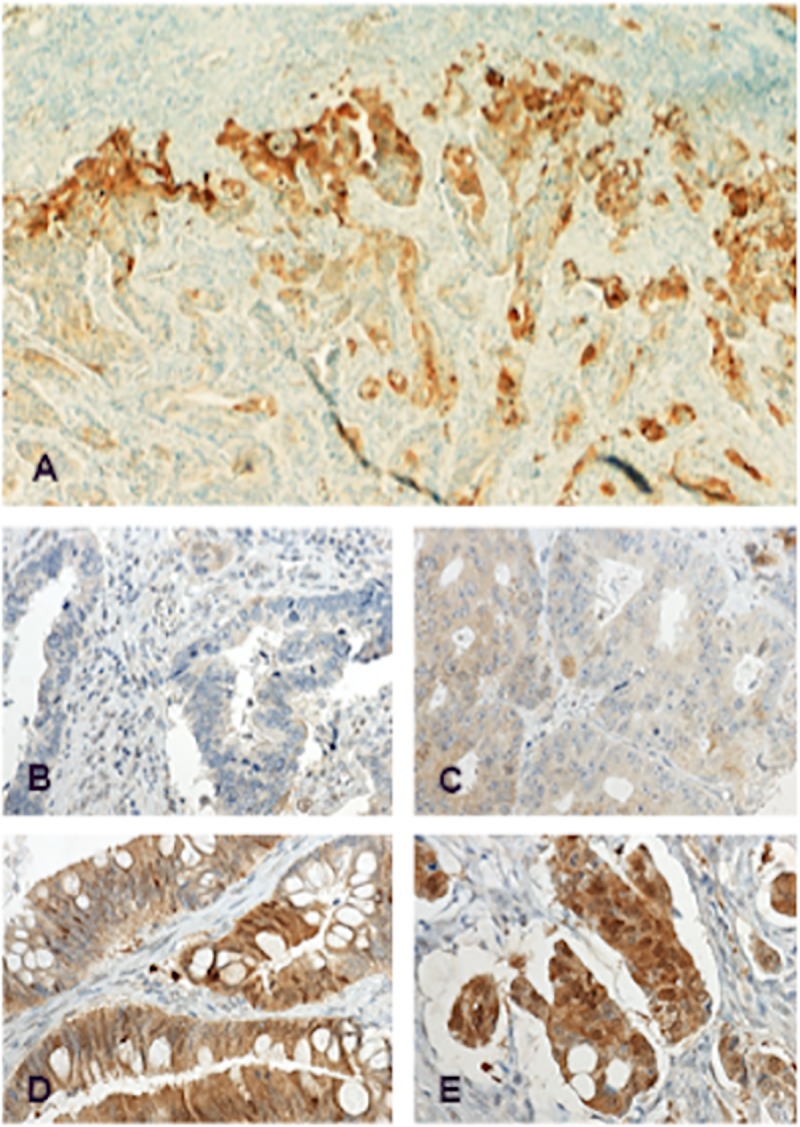
Immunohistochemical staining patterns of AZIN2 in colorectal cancer. Representative images of AZIN2 expression in colorectal cancer, A) strong positivity in the invasive front, B) negative AZIN2 expression, C) low positivity, D) moderate positivity, E) strong positivity. Original magnification x50 (A) and x400 (B-E).

AZIN2 cytoplasmic expression in the tumor cells was scored as negative-low-moderate-high according to intensity. For statistical analysis, tumors were grouped into low (negative to low) and high (moderate to high). The stainings were scored independently by T.K. and J.H., who were blinded to the clinical data and outcome. Any differences in scoring were discussed until consensus. Fig 1 shows the representative images of expression. An Eclipse 80i microscope was used, images were taken by a DS-Fi1camera using NIS Elements software, all by Nikon (Nikon Corporation, Tokyo, Japan).

### Statistical analyses

The association between AZIN2 expression and clinicopathological parameters was evaluated using the exact Pearson Chi-square test or the exact linear-by-linear association test for ordered parameters. DSS was counted from date of surgery to date of death from colorectal cancer, or until end of follow-up. Survival analysis by the Kaplan-Meier method was compared using the log rank test. The Cox regression proportional hazard model, adjusted for sex, age, Dukes classification, and differentiation, served for uni- and multivariable survival analysis. Testing of the Cox model assumption of constant hazard ratios over time included a time-dependent covariate separately for each tested variable. The hazard ratio of differentiation was analyzed in two periods (0–1.25 and 1.25–5 years) in order to meet the assumptions of the Cox model, with the time-dependent Cox model. Interaction terms were considered but none were found. All tests were two-sided. A p-value of 0.05 was considered significant. All statistical analyses were done with the SPSS version 20.0 (IBM SPSS Statistics, version 20.0 for Mac; SPSS, Inc., Chicago, IL, USA).

## Results

### Immunohistochemistry

Immunohistochemistry revealed a slightly granular distribution of the cytoplasmic AZIN2 in the tumor cells. The strongest AZIN2 expression was seen in the invasive cells of the tumor front. Cells with robust AZIN2 expression displayed morphological features of epithelial-mesenchymal transition (EMT) (Fig 1). AZIN2 staining could be evaluated in 694 of the 840 tumors represented in the TMA; 81 (11.7%) were scored as negative, 263 (37.9%) as low, 257 (37.0%) as moderate, and 93 (13.4%) as high expression.

### AZIN2 and clinicopathological parameters

High cytoplasmic AZIN2 expression was associated with mucinous histology (p = 0.002) and location in the right hemicolon (p = 0.021). The histological grade of the tumor did not correlate with AZIN2 expression. No association emerged with age, gender, stage, or location (colon vs. rectum) (Table 1).

**Table 1:** Association between AZIN2 expression and clinicopathological parameters.

AZIN2 Expression			
	Negative-low	moderate-high	
n(%)	344 (49.6)	350 (50.4)	p-value*
**Age, years**			
<65	152 (44.2)	151 (43.1)	0.818
≥65	192 (55.8)	199 (56.9)	
**Gender**			
Male	158 (45.9)	145 (41.4)	0.251
Female	186 (54.1)	205 (58.6)	
**Dukes**			
A	52 (15.1)	47 (13.4)	0.370
B	123 (35.8)	125 (35.7)	
C	96 (27.9)	92 (26.3)	
D	73 (21.2)	86 (24.6)	
**Grade (WHO)**			
1	13 (3.8)	10 (2.9)	0.442
2	242 (71.2)	245 (70.0)	
3	75 (22.1)	83 (23.7)	
4	10 (2.9)	12 (3.4)	
Missing	4	0	
**Location**			
Colon	169 (49.1)	183 (52.3)	0.448
Rectum	175 (50.9)	167 (47.7)	
**Side**			
Right	80 (23.3)	109 (31.1)	0.021
Left	264 (76.7)	241 (68.9)	
**Histology**			
Non-mucinous	325 (94.5)	306 (87.7)	0.002
Mucinous	19 (5.5)	43 (12.3)	

* By the exact Pearson Chi-square test or the exact linear-by-linear association test for ordered parameters

### Survival analysis

**Fig 2:**
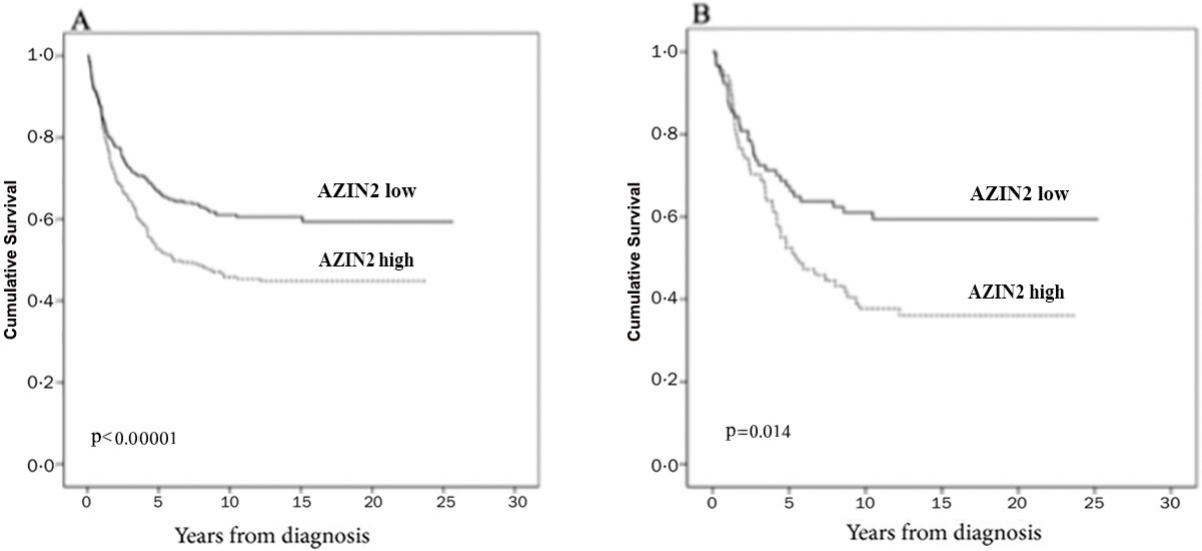
High expression of AZIN2 is associated with poor prognosis. Disease-specific survival analysis according to the Kaplan-Meier method for AZIN2 expression in (A) colorectal cancer, (B) Dukes C colorectal cancer. Log-rank test was the test used.

High AZIN2 expression in CRC was a sign of unfavorable prognosis (p<0.0001, log-rank test); 5-years DSS for patients with high AZIN2 tumor expression was 52.5% (95 CI 42.0–58.0) compared to 66.9% (95% CI 52.9–72.1) for those with low expression. When we stratified CRC according to stage, high AZIN2 expression was a sign of unfavorable prognosis in Dukes C (Stage III) CRC (p = 0.014); 5-years DSS for patients with high AZIN2 tumor expression was 52.4% (95 CI 41.4–63.3) compared to 67.5% (95% CI 57.5–77.5) for those with low expression. (Fig 2)

These results were confirmed by 5-year univariable Cox regression analysis for CRC. Cox regression multivariable analysis adjusted for age, gender, sex, stage, and histological grade showed that high AZIN2 expression was an independent factor of an unfavorable prognosis in CRC. (Table 2)

**Table 2:** Cox uni-and multivariable analysis of relative risk of death from colorectal cancer within 5 years by AZIN2 expression.

AZIN2 expression	HR (95% CI)	P-value	N (events)	HR (95% CI)	P-value	N (events)
	Univariable			Multivariable		
Low	1.00		344(122)	1.00		340(120)
High	1.23(1.09–1.40)	0.001	350(172)	1.61 (1.20–2.32)	0.003	350 (172)

CI = confidence interval, HR = Hazard ratio. Multivariable analysis included adjustment for gender, Dukes class, differentiation grade (G1/2 vs G3/4).

### Increased expression of AZIN2 follows the induction of EMT in HT-29 cells

**Fig 3:**
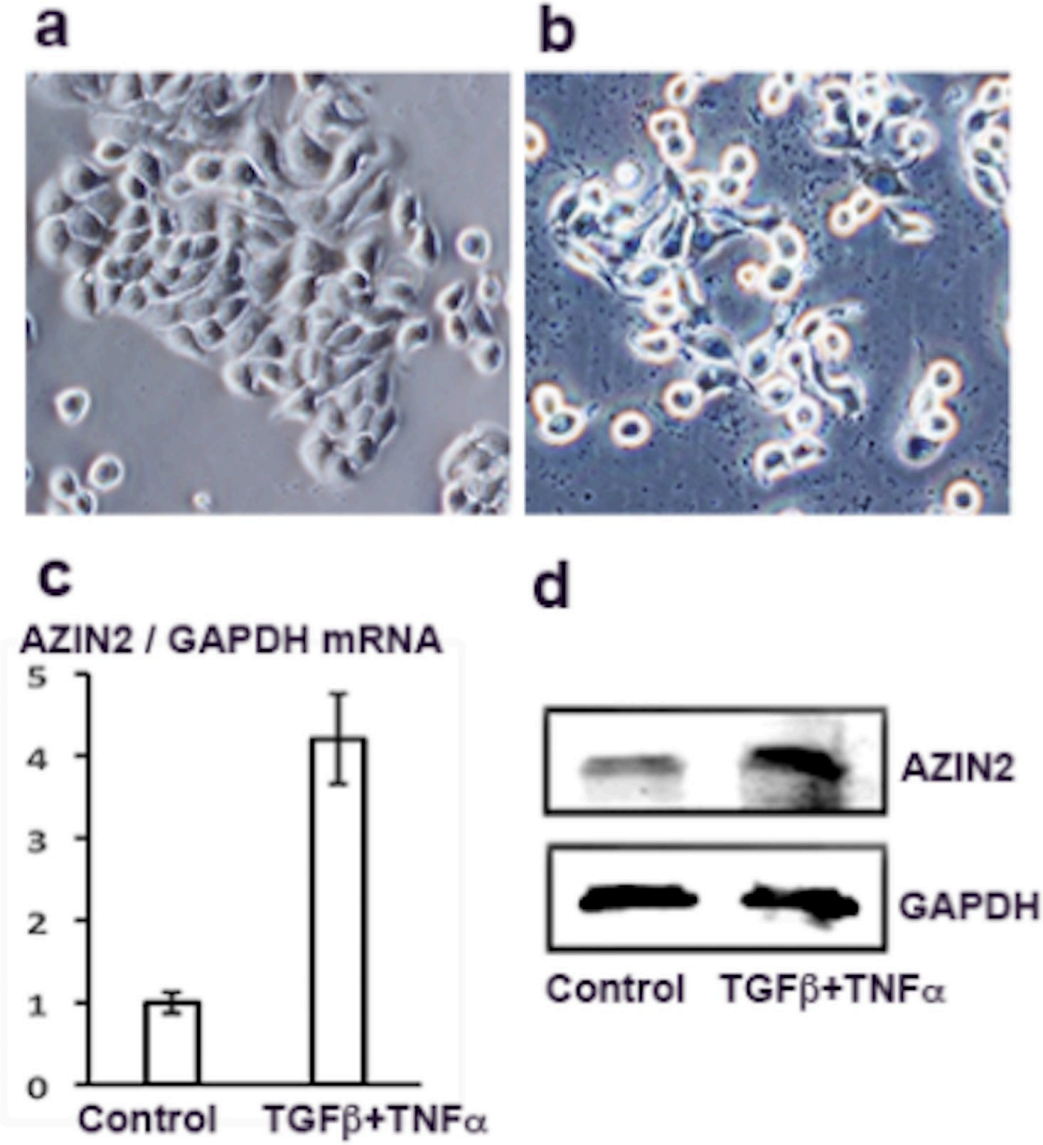
Increased expression of AZIN2 follows the induction of EMT in HT-29 cells. HT-29 cells (a) after treatment with TGFβ+TNFα for 48 h (b) show upregulated expression of AZIN2 transcript (c) and AZIN2 protein (d). Panel c shows the results of four RT-qPCR experiments with error bars indicating SD. Panel d shows Western blotting with K3 antibodies.

Treatment of HT-29 cells cultured for 48 h with a combination of TGFβ and TNFα induced morphological EMT (Fig 3A and 3B) and upregulated expression of AZIN2 mRNA (Fig 3C) and AZIN2 protein (Fig 3D). A similar increased expression of AZIN2 was also seen in LS174T cells and in A549 cells after treatment with TGFβ and TNFα (S1 Fig). The impact on the expression of EMT marker proteins after treatment of A549, HT29, and LS174T cells with TGFβ and TNFα for four days are summarized in S1 Table.

### Enhanced release of CD63-positive exosomes from T84 colon cancer cells over-expressing AZIN2

**Fig 4:**
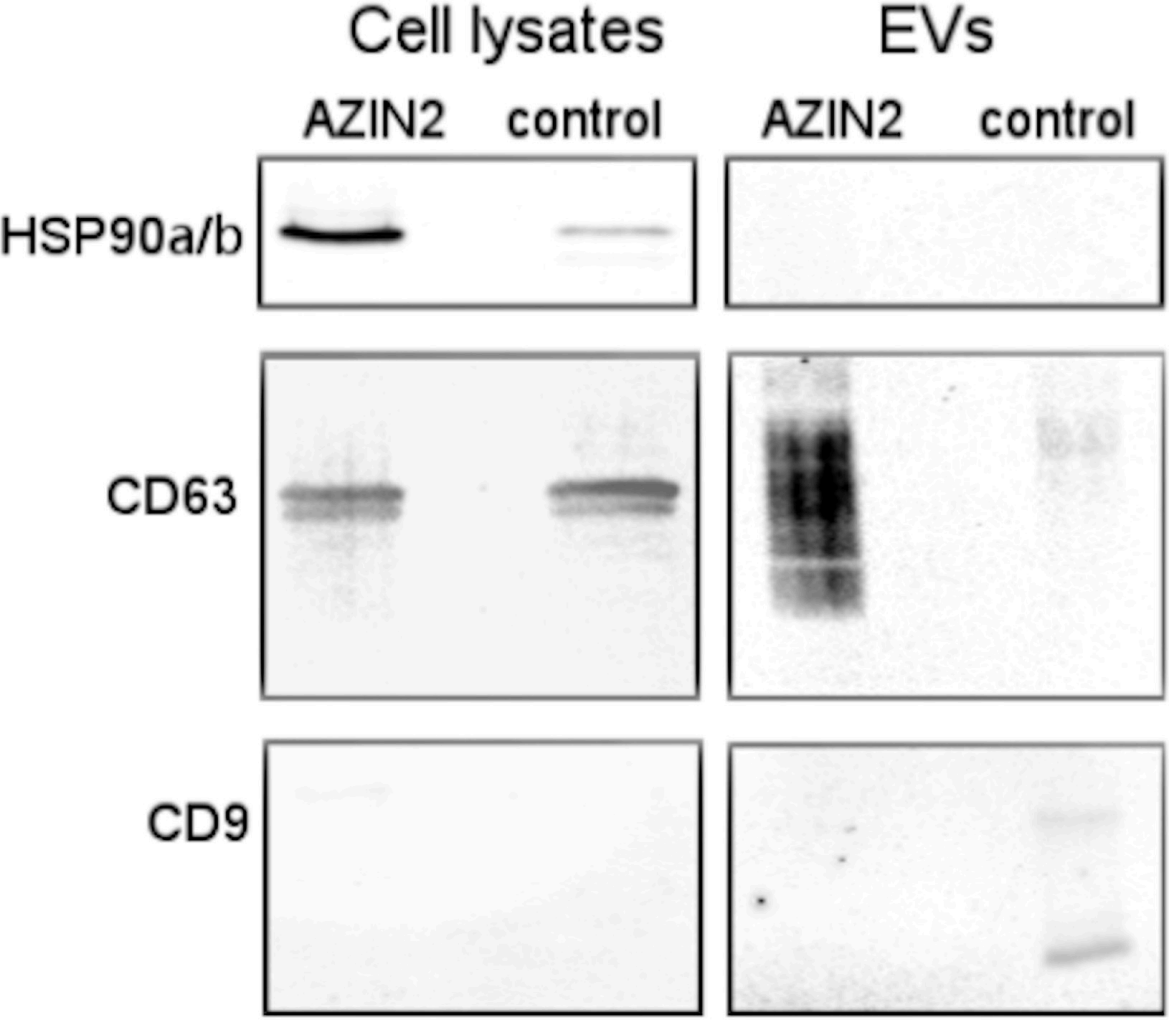
AZIN2 over-expressing T84 colon cancer cells release CD63 positive exosomes. Immunoblotting of cell lysates (15μg) and of exosomes (EV) (2 μg) isolated by ultracentrifugation from culture supernatant of AZIN2 cDNA over-expressing and control T84 colon cancer cells transfected with the empty vector using antibodies to HSP90a/b, CD63 and CD9.

Over-expression of human AZIN2 cDNA in T84 colon cancer induced accumulation of CD63-positive exosomes in the culture medium. On the other hand, the amount of CD9-positive material decreased in the culture medium of AZIN2-expressing T84 cells, compared with medium from cells transfected with the empty vector. The exosome markers CD63 and CD9 were highly enriched in exosomes compared with cell lysates. HSP90α/β, which is a marker for intracellular protein, was absent from purified exosome preparations but present in cell lysates (Fig 4).

## Discussion

Cancers typically display increased catalytic activity of ODC and elevated concentrations of polyamines. AZIN1 and AZIN2 are both positive activators of ODC. AZIN1, which is physiologically involved in regulating the cell cycle and in stimulating cell proliferation, has been associated with cancer. Furthermore, it has been identified as a predictive factor of relapse in pediatric pre-B ALL [22] and associated with aggressive behavior in cancers of the breast, prostate, lung, and ovary [23].

There are no previous reports on the impact of AZIN2 on human cancer. Here we show that increased tumor expression of AZIN2 is an independent predictor of unfavorable prognosis in CRC. Immunohistochemical stainings of sections from colon cancers revealed particularly strong AZIN2 expression in invasive cells of the tumor front, showing morphological features of EMT. Enhanced AZIN2 expression was also found in cancers with mucinous histology.

The molecular details of the mechanisms that link elevated AZIN2 expression to poorer prognosis in CRC are still unclear. It appears, however, that the effect is not directly linked to the rate of cancer cell growth. There was no correlation between the histological grade, i.e. level of differentiation and AZIN2 expression. Moreover, the highest physiological expression of AZIN2 is found in functionally differentiated and slowly proliferating cells with active vesicle transport or secretory features [12].

Many cancers have been found to acquire a secretory phenotype that associates with poorer outcome [24]. This may occur via senescence-associated secretory phenotype or EMT-associated secretory phenotype. A common feature is increased extracellular release of secrotomes containing bioactive molecules that modify the local microenvironment and stimulate intercellular cross-talk. Elevated expression of the Rab27 family of small GTPases that regulate vesicle exocytose has also been found to associate with adverse prognosis in cancer of the breast, pancreas, bladder and colon [25–28]. The expression levels of Rab27 GTPases were however not investigated in this material.

Our observation of enhanced expression of AZIN2 in infiltrating cancer cells with morphological features of EMT, defined in [29], impelled us to investigate whether induced acquisition of a mesenchymal phenotype associates with up-regulated levels of AZIN2. By using the previously described in vitro model with the colon carcinoma cell line we confirmed that induction of EMT in culture indeed did cause elevated expression levels of AZIN2 mRNA and protein. The molecular pathways regulating the enhanced AZIN2 expression in relation to EMT are under investigation like also the question of whether forced overexpression of AZIN2 directly induces EMT in cancer cells.

We found that over-expression of AZIN2 in T84 CRC cells induced accumulation of cell-derived CD63-positive vesicles in the culture medium, indicating functional features of a secretory phenotype. Expression of the exosome marker CD9, which we also investigated, was down-regulated in vesicles derived from cells over-expressing AZIN2 compared to cells transfected with the empty vector. This may be of some significance since expression of CD9 in CRC has been found to correlate with a more favorable prognosis, whereas loss of CD9 correlates with a poorer prognosis [30].

Our findings of enhanced exosome release in vitro from cancer cells with high AZIN2 expression provide an explanation to the correlation between AZIN2 levels and prognosis in CRC. Since our TMA study was carried out on an archival material we have no information available on the in vivo exosome activity in individual cases. Therefore the role of the concentrations of tumor-derived exosomes in the body fluids of CRC patients with cancers showing high AZIN2 expression needs to be investigated further.

AZIN2 expression was higher in cancers arising from the right colon compared to left colon, which may be due to the higher frequency of tumors with mucinous histology in the proximal colon. The elevated expression of AZIN2 in CRC with mucinous histology is in line with the functional role of AZIN2 as a regulator of intracellular vesicle transport facilitating mucin secretion.

Given our findings of high AZIN2 expression in cancer cells with features of EMT, it is conceivable that AZIN2 constitutes a signature for EMT-associated secretory phenotype–a feature that has been found to predict poorer cancer survival [31].

## Supporting information

S1 Fig **Treatment of A549 and LS174T cells with TGFβ+TNFα for three days induces EMT morphology (a) and elevated expression of AZIN2 (b)**. Loss of E-cadherin was seen in A549 cells (b).
(TIFF)

S1 Table Induction of EMT by TGFβ and TNFα in different cell lines and effect on protein expression. (DOCX)
