# Supplementary figures and images for: Ornithine decarboxylase antizyme inhibitor 2 (AZIN2) is a signature of secretory phenotype and independent predictor of adverse prognosis in colorectal cancer
Source: PLoS One. 2019 Feb 15;14(2):e0211564. doi: 10.1371/journal.pone.0211564 (PMC6377119; doi:10.1371/journal.pone.0211564)

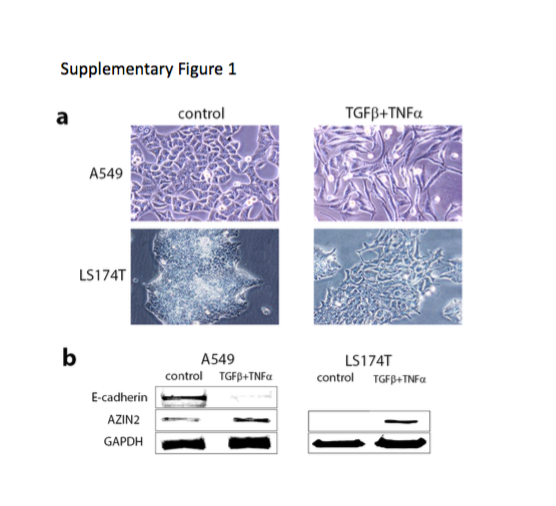

Supplement: S1 Fig — Treatment of A549 and LS174T cells with TGFβ+TNFα for three days induces EMT morphology (a) and elevated expression of AZIN2 (b). Loss of E-cadherin was seen in A549 cells (b). (TIFF) [file pone.0211564.s001.tiff]
