# Supplementary material for: Ornithine decarboxylase antizyme inhibitor 2 (AZIN2) is a signature of secretory phenotype and independent predictor of adverse prognosis in colorectal cancer
Source: PLoS One. 2019 Feb 15;14(2):e0211564. doi: 10.1371/journal.pone.0211564 (PMC6377119; doi:10.1371/journal.pone.0211564)
Supplement: S1 Table — (DOCX) [file pone.0211564.s002.docx]

**Supplementary Table 1 Induction of EMT by TGFβ and TNFα in different cell lines and effect on protein expression**

|  | **E-cadherin** | **Vimentin** | **Fibronectin** | **AZIN2** |
| --- | --- | --- | --- | --- |
| **A549** | ↓ | ↑ | ↑ | ↑ |
| **HT29** | NC | ↑ | NC | ↑ |
| **LS174T** | NC | ↑ | NC | ↑ |

↓ = decreased expression of the protein detected by western blot

↑ = increased expression of the protein detected by western blot

NC = no change in the expression of protein detected by western blot
